# Supplementary material for: Noise reduction and quantification of fiber orientations in greyscale images
Source: PLoS One. 2020 Jan 16;15(1):e0227534. doi: 10.1371/journal.pone.0227534 (PMC6964846; doi:10.1371/journal.pone.0227534)
Supplement: S1 Table — (PDF) [file pone.0227534.s001.pdf]

$\Delta_{\mathrm{MC}}[\%]$	*Lena* 512×512	*Boat* 256×256	*Cameraman* 512×512
total deviation	0.02 ± 2.15	0.01 ± 3.10	0.01 ± 2.26
without boundary	-0.16 ± 0.44	-0.36 ± 0.62	-0.17 ± 0.72
boundary	23.11 ± 5.54	23.32 ± 6.33	22.73 ± 8.18
