# Supplementary material for: Noise reduction and quantification of fiber orientations in greyscale images
Source: PLoS One. 2020 Jan 16;15(1):e0227534. doi: 10.1371/journal.pone.0227534 (PMC6964846; doi:10.1371/journal.pone.0227534)
Supplement: S2 Table — (PDF) [file pone.0227534.s002.pdf]

$\Delta_{\mathrm{MC}}[\%]$	*Lena* 512×512	*Boat* 256×256	*Cameraman* 512×512
total deviation	59.32 ± 26.48	36.97 ± 25.11	53.06 ± 28.32
$\delta_{\mathrm{cut}} = 100\%$	-7.83 ± 5.51	-8.09 ± 5.46	-7.97 ± 5.47
$\delta_{\mathrm{cut}} = 50\%$	-2.44 ± 1.70	-2.54 ± 1.73	-2.97 ± 1.68
$\delta_{\mathrm{cut}} = 10\%$	-0.22 ± 0.16	-0.22 ± 0.16	-0.21 ± 0.16
