# Supplementary material for: Noise reduction and quantification of fiber orientations in greyscale images
Source: PLoS One. 2020 Jan 16;15(1):e0227534. doi: 10.1371/journal.pone.0227534 (PMC6964846; doi:10.1371/journal.pone.0227534)
Supplement: Attachment — Submitted filename: Letter_Reviewer2.docx [file pone.0227534.s007.docx]

Dear Reviewer,

thank you a lot for your time and effort to provide such detailed and constructive criticism on our submission:

***„Noise Reduction and Quantification of Fiber Orientations in Greyscale Images“***

We found that your remarks were very helpful for us to clarify the benefits of our method. We have followed your valuable advice and are now resubmitting our improved manuscript.

In the following we provide responses to your comments:

*1) The effect of edge on the FFT spectrum is well known just as how to remove it (Moisan 2011) so it is not necessary to repeat this analysis. Simply state you removed the edge artifacts according to Moisan 2011 and remove the comparison (Figure 2) since it is not new.*

We agree that the edge artefact removal is not new, but we found that it is rarely used in quantitative calculations involving the Fourier transform. Therefore, we would like to visually emphasize the benefits coming from the periodic decomposition in comparison to commonly used window function.

*2) L156 is the block 3x3 enough to remove the effect of mean intensity value of the image which does not carry relevant information? The sensitivity analysis should be performed here to justify Author´s choice.*

We performed a sensitivity analysis on different cut-off radii, which we will provide in the supplements. It is also shown in Figure 1

Figure 1: Sensitivity analysis. (A) Squared difference between calculated angular orientation distribution and sampled fiber angle distribution on N=10,000 images (Monte-Carlo generated). The same dataset was also used for evaluation parameter optimization. Unfiltered power spectra are used to isolate the effect of the cut-off radius. (B-D) Evaluation example for cut-off radii 0,3, and 6 pixels. The artifact dramatically reduces from a 0 cut-off to a radial cut-off of 3 pixels. Cut-off radii above 3 pixels barely influence the angular distribution. In order to save angular information, we choose a cut-off radius of 3 pixels.

Indeed, the sensitivity analysis of an unfiltered power spectrum revealed that a 3x3 block (cut-off radius = two) is not optimal to remove the artifacts. We choose a cut-off radius of three pixel, which is equal to a 5x5 block to remove the effect.

*3) The weighting parameter alpha seems to be just some sort of weighting function similar to one proposed before (Polzer et al. 2013) so it should be discussed what is the advantage of proposed approach over the already published one.*

Polzer et. al (2013) introduced a weighting factor which raises the whole orientation distribution. Our weighting factor only amplifies the number of remaining pixels (after filtering) within a certain angular interval of the power spectrum. We now explain the difference between both weighting factors in the discussion of our manuscript. In addition, we think that our weighting function is be less dependent on the fiber properties and image quality. We found that the optimal estimation of our weighting factor $\alpha$ is quite stable ($1<\alpha<2$) throughout different noise factors and fiber geometries. We provide a plot of the optimal evaluation parameter $\delta_{Cut}$ and $\alpha$ vs. dispersion (k), noise factor (NF), aspect ratio (AR) and width in the supplement (S2 Fig).

*4) I do not understand the figure 6. Authors claimed the sigmoid like function can fit the data and be pi-periodic (eq.19) but the figure 6 does not show such periodicity at all since values in 0° and 180° does not match. Please explain that.*

We agree that the figure caption was misleading. We wanted to point out, that our modified sigmoid function models the cumulative distribution function of a pi-periodic signal, which is not given by a simple sigmoid function. Since figure 6 was very confusing to both reviewers we now excluded it from the manuscript.

*5) I do not understand the reasoning behind performing your comparisons on test images (boat, Lena) which are commonly used in the image analyses but have pretty much nothing in common with the images of tissues which your algorithm should be used for.*

We wanted to clarify that our filtering algorithm can be applied to any kind of image. We think that it is easier for the reader to interpret the effects of a filtered Fourier transform concerning the effects on the back-transformation and the loss of image information (figure 4).

*6) Why do the Authors chose to filter the power spectrum? I know it is possible but I failed to see what would be an advantage of doing so? Moreover how can one estimate such uncertainty (necessary for the threshold estimation) in real images where the noise function is not known? Is that even possible?*

Filtering the power spectrum does improve the accuracy of the angular orientation distribution, which was also shown by band-pass filtering (Morrill et. al, 2017). We developed a general filter, which is adaptive to the underlying signal and not depended on the fiber geometry. We now showed the improved accuracy for different fiber geometries with respect to band-pass filtering (figure 6 + figure 7).

We think that the estimated parameter are applicable to different scientific images as long as an image error, like the photon counting error $\surd N$ can be assumed. We did prove that by applying our method to *in vivo* SHG (second harmonic generation) images of dermal collagen (figure 8 + figure 9).

*7) L126 why does the lengths of fibers follow normal distribution? Can Authors provide any evidence for that?*

The distribution of fiber lengths is not relevant to the image analysis. We have now modified our Monte-Carlo procedure and created images containing fibers of a single width and length. We additionally used to analyze the influence of fiber geometry on the accuracy of the algorithm.

*And what of the image has longer fibers aligned preferentially in one direction and shorter ones in the other. Can their algorithm still capture the distribution of such fibers correctly?*

That is indeed a very exciting scenario that we would like to analyze. We hope that we can face such scenarios in the future.

*8) Why Authors did not apply their algorithm on real images of real tissues? There is no problem in estimating to correctly estimate The problems arise from analysis of the real tissues.*

We first wanted to confine our manuscript to the methodological part and provide experimental results in subsequent publications. We very much agree with you, that the benefits and the applicability of our algorithm on real images needs to be shown.

Therefore we now provide an exemplary application, *in vivo* SHG images of human dermal collagen to demonstrate that our algorithm can accurately predict the fiber orientation in SHG images which potentially suffer from a low image quality (figure 8 and figure 9 and the corresponding text).

We appreciate the effort and time you invested in reviewing our paper and are thankful for all your suggestions. Furthermore, we are sorry that we can not elaborate on all your suggestions for our manuscript due to length constraints. We are confident that the new manuscript answers most of your questions.

Thank you in advance.

Yours faithfully,

Maximilian Witte, Frank Fischer, Michael Rübhausen
