# Supplementary material for: Noise reduction and quantification of fiber orientations in greyscale images
Source: PLoS One. 2020 Jan 16;15(1):e0227534. doi: 10.1371/journal.pone.0227534 (PMC6964846; doi:10.1371/journal.pone.0227534)
Supplement: Attachment — Submitted filename: Response_to_Reviewer.docx [file pone.0227534.s008.docx]

Dear Reviewers,

we appreciate your time and effort to reconsider our manuscript:

***„Noise Reduction and Quantification of Fiber Orientations in Greyscale Images“***

Thank you again for your beneficial comments. In the following we are providing responses to each of your comments:

Major comment (summarized):

*Use preprocessed (periodic plus smooth decomposition) images for the comparison of the band-pass method with the AF method.*

We fully agree with your point. We noticed, that our generated MC images rarely suffer from any cross-like artifacts. However, we re-calculated the errors and levels of significance using the smooth component of the decomposition for the band-pass method. The global error of the dispersion coefficient k sligthly decreased from $\left( 34.3\pm26.7 \right)\%$ to $\left( 33.9\pm26.5 \right)\%$.

Other comments:

*- In the original submission, figure 8 was included to show the advantage of using a Sigmoid function vs Von-Mises function. It appears that this was removed, and there is no longer any quantitative or qualitative comparison with a Von-Mises function. Therefore, the advantage of a Sigmoid function is no longer apparent w.r.t. a conventional Von-Mises approach. A figure showing the difference in error using these two different methods should be returned to the paper (could be a supplemental figure).*

We agree on that. We adjusted the figure to the current dataset and added it to our supplement (S3_Fig). In addition, we refer to the supplemental figure in the discussion of the revised manuscript.

*- For calculating fiber dispersion error for the AF method, please make it clear how you calculated percent error between the parameter (k) input into the von-Mises function to generate the test images, and the parameter (b) that was obtained by fitting the sigmoid function.*

As stated in our manuscript (lines 228-232) we fitted both, the frequency distribution and the cumulative frequency distribution of the sampled fiber angles to obtain reference parameter b and k. Using this approach, we additionally account for statistical sampling errors. If we use the predefined value of k as reference value we get an error of $\left( 35.0\pm25.6 \right)\%$ as global error for the band-pass method (preprocessed images).

*- Please include the magnification of the SHG images, and include a scale bar on the images in Fig. 8.

- For transparency, all generated images used to compare the AF method and the band-pass method should be made publicly available via a public repository.*

We also included a scale bar on the images and uploaded the data to a figshare repository.

Yours faithfully,

Maximilian Witte, Frank Fischer, Michael Rübhausen
